# Non-healing old world cutaneous leishmaniasis caused by *L. infantum* in a patient from Spain

**DOI:** 10.1186/1471-2334-14-206

**Published:** 2014-04-16

**Authors:** Arne Kroidl, Inge Kroidl, Gisela Bretzel, Thomas Löscher

**Affiliations:** 1 Division of Infectious Diseases and Tropical Medicine, Medical Center of the University of Munich, Leopoldstr. 5, 80802 Munich, Germany

**Keywords:** Cutaneous leishmaniasis, *Leishmania infantum*, surgical treatment

## Abstract

**Background:**

The prevalence of Old World Cutaneous Leishmaniasis in the Mediterranean region is increasing and in Southern Europe often caused by *Leishmania infantum.* Spontaneous healing of cutaneous leishmaniasis is commonly observed, especially if caused by *L. major,* whereas *L. infantum* associated lesions have been reported with longer disease duration and decreased tendency for self-limitation, however, available information is sparse.

**Case presentation:**

We report the case of an otherwise healthy woman from Southern Spain who presented with a seven years persistent, non-healing, painless, central ulcerated, nodular cutaneous lesion with a diameter of 2 cm of the forearm. Cutaneous leishmaniasis was diagnosed by smear and histology, showing large amounts of leishmania amastigotes in subepidermal histiocytes and extensive lymphocyte and plasma cell inflammation. *L. infantum* as the causative pathogen was confirmed by restriction fragment length polymorphism and microsatellite-PCR. Systemic or visceral involvement was excluded by negative leishmania serology and clinical presentation, relevant concomitant diseases or immunosuppression were excluded including quantification of immunoglobulin levels and lymphocyte phenotyping. Topical and systemic anti-infectious treatment options, often limited in terms of efficacy, tolerability and long lasting treatment duration, were considered. Treatment was successfully performed by surgical extraction in local anaesthesia only.

**Conclusion:**

To our knowledge this is the longest reported duration of a *L. infantum* associated cutaneous leishmaniasis indicating a potential long lasting natural evolution of the disease in an otherwise healthy and immunocompetent patient, however, high parasite density may have reflected a lack of a *L. infantum* specific immune response. Complete surgical extraction can be successfully performed as treatment.

## Background

The prevalence of cutaneous leishmaniasis (CL) in the Mediterranean region is increasing and the variety of causative leishmania species is diversifying possibly due to worldwide travel and migration, increase of animal reservoirs, and climate change favouring the spread of the phlebotomine sandfly vectors [1]. Mediterranean Old World CL (OWCL) can be caused by three different Leishmania species. In Europe, CL is usually associated with zoonotic *Leishmania infantum* infection with domestic dogs as a main reservoir. CL caused by anthroponotic *L. tropica* is common in Western and Southern Asia and has been reported from Greece but not from other parts of Europe [1,2]. *L. major* infection has a zoonotic reservoir in rodents and is absent in Europe but a common cause of OWCL in North Africa, Middle East and some regions in Subsaharan Africa [1]. High prevalence rates of *L. infantum* in asymptomatic human carriers from Southern Europe indicate that the disease might be of greater public health relevance [3] as *L. infantum* can also lead to life threatening visceral leishmaniasis (VL) especially in individuals with underlying immunosuppression such as patients with HIV infection. Spontaneous healing of OWCL is commonly observed after several months, especially if caused by *L. major*[1,2,4], and has been associated in animal models with the development of specific T helper cell Th1 immune responses [5]. *L. infantum* associated OWCL seems to have longer disease duration associated with a decreased tendency for self-limitation, however, available information is sparse.

## Case presentation

**Figure 1:**
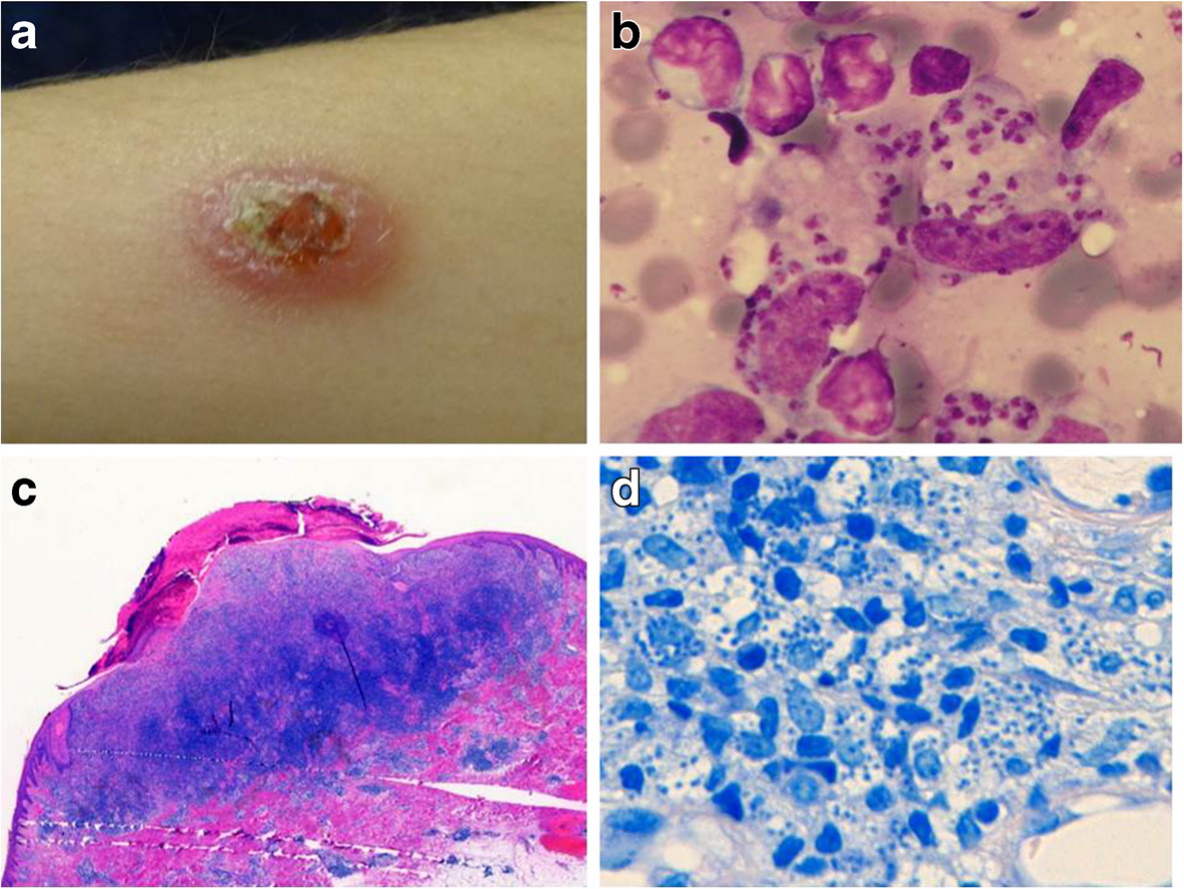
**Clinical presentation and histology of the *Leishmania *lesion. (a)** Ulcerated, nodular lesion on the left forearm, **(b)** Leishmania amastigotes in a biopsy smear (Giemsa stain), **(c)** excised ulcer with subepidermal lymphocyte and plasma cell inflammation (HE stain), **(d)** large amounts of Leishmania amastigotes in histiocytes (Giemsa stain).

We report the case of a 35 year-old female who presented in January 2013 with a single painless, erythematous, central ulcerated, nodular lesion with a diameter of approximately 2 cm on her left forearm (Figure 1a). The patient has a Moroccan family background, but was born and grew up in Germany, and moved in 2006 to the region of Granada in Spain. Within the same year a cutaneous lesion started to appear as an erythematous spot which developed over the following seven years to the current presentation in the absence of systemic symptoms such as fever, weight loss or fatigue. Various topical treatments were without significant effect, swab cultures from the ulcer showed only bacteria of the normal skin flora. We could confirm the diagnosis of OWCL from a small biopsy sample by positive smears (Figure 1b), and histology revealed large amounts of leishmania amastigotes in subepidermal histiocytes and extensive lymphocyte and plasma cell inflammation (Figure 1c and d). PCR diagnostic showed a restriction fragment length polymorphism (RFLP) pattern of *L. donovani/L. infantum complex* in an in-house assay [6]. Further differentiation was performed at a German reference center (Dr. Gabriele Schönian, Institute of Microbiology and Hygiene, Humboldt University, Charité, Berlin) using microsatellite-PCR (Marker LIST7039) [7] confirming *L. infantum* as the causative pathogen. Systemic involvement or visceral leishmaniasis was excluded by negative serological markers (ELISA, IFT, immunoblot) using *L. infantum* as antigen, absence of systemic clinical symptoms including absence of lymphadenopathy or hepatosplenomegaly in ultrasound diagnostic. Concomitant disease or immunosuppression were excluded by laboratory parameters including normal values for haematology, clinical organ chemistry, plasma protein and quantitative immunoglobulin levels, normal absolute and relative values in lymphocyte phenotyping for T-cell, B-cell and NK cell populations, negative serological testing for HIV, syphilis, hepatitis B and C. No other skin lesions were present, sandfly exposure and family history of similar lesions was not obtained. Although, the patient regularly travelled to Morocco the infection was probably acquired in Spain since the last travel to Morocco had been more than 1 year before onset

After a thorough discussion with the patient about therapeutic alternatives such as cryotherapy, topical antimony therapy, or systemic chemotherapy, it was decided to perform a total excision of the lesion in local anaesthesia, taking into consideration its small size and long duration as well as an imminent business trip of the patient. Follow-up visits after 11 months showed complete healing with a minimal residual scar.

## Conclusion

This case represents to our knowledge the longest reported duration with seven years persistence of a *L. infantum* CL indicating a potential long lasting natural evolution of the disease in an otherwise healthy and immunocompetent patient. OWCL caused by *L. major* usually heal within 2–8 months, and lesions in *L. tropica* infection often heal spontaneously within one year [4]. Cases of chronic, non-healing cutaneous or mucocutaneous leishmanisis caused by *L. infantum* have been reported mostly in patients with HIV or immunosuppressive treatments, or in patients with atypical clinical presentations such as relapsing CL, leishmaniasis recidivans (LR) or mucosal localizations [8,9]. A maximum duration until spontaneous healing of up to 3 years has been reported for typical *L. infantum* OWCL lesions in immunocompetent patients [9]. In Turkey, Cukurova region, a CL focus initially linked to *L. infantum* by PCR-RFLP, transmitted by *Phlebotomus tobbi* was described and patients presented mostly with small, non-ulcerative lesions which lasted for at least 2 years with only few cases of spontaneous healing [10]. As in our case negative leishmania serology was seen in these patients which is probably typical for CL cases caused by *L. infantum*. Recently the causative *Leishmania* strain from this region was revealed by whole genome sequencing as a natural hybrid between *L. infantum* and *L. donovani*[11], indicating that diagnostic molecular methods might have some limitation for species differentiation. Unfortunately, we were not able to isolate the parasite in our case into culture for further specification and origin detection.

As reviewed by Peters and Sacks [5] chronic or progressive leishmaniasis has been associated by the parasite’s ability to evade macrophage defense mechanism such as killing by oxygen-derived free radicals, to produce defects in the induction and expression of cell-mediated immune mechanisms such as suboptimal T-cell receptor (TCR) signaling and the inability of *Leishmania*-infected macrophages to produce IL-12 as the main inducer of IFN-y and CD4+ T cell Th1 differentiation resulting in defected cellular immune response. Furthermore it was speculated that the regulation of adaptive immune responses by CD25 + Foxp3+ T regulatory cells in the skin downregulate parasite-specific immunity allowing *Leishmania* species to establish chronic infection. It is speculative if such mechanism applied in our case, either caused by the host’s local immune characteristics or special phenotypic properties of the parasite itself – such as the hybrid strain from the Cukurova region - evading immune control. Lack of immune control in our case might be supported by high parasite density seen in histology and smear.

For OWCL, several topical and systemic treatment options are available. However, all have substantial limitations in terms of efficacy and tolerability [4]. For *L. infantum* CL there are currently no randomized, placebo-controlled treatment studies available to guide treatment decision. Small observational studies and case reports have described treatment success with intralesional antimonials, cryotherapy or oral triazole antifungals [12-14]. Other systemic treatment options such as pentavalent antimony, pentamidine, amphotericin B or miltefosine should be reserved for complex or complicated disease because of the risk of significant toxicity. In uncomplicated OWCL presenting as a single lesion of limited size (<4 cm of diameter) surgical excision has also been applied successfully [15], which was also confirmed in our case.

## Consent

Written informed consent was obtained from the patient for publication of this case report and any accompanying images. A copy of the written consent is available for review by the Editor of this journal.

## Competing interests

The authors declare that they have no competing interests.

## Authors’ contributions

AK wrote the manuscript, lead patient management, data collection, analysis and discussion of results; IK participated in data collection and discussion; GB lead diagnostic laboratory procedures, TL suggested important intellectual content, discussion of results and participated in manuscript writing and review. All authors read and approved the final manuscript.

## Pre-publication history

The pre-publication history for this paper can be accessed here:

http://www.biomedcentral.com/1471-2334/14/206/prepub

## References

[B1] Antoniou M Gramiccia M Molina R Dvorak V Volf P The role of indigenous phlebotomine sandflies and mammals in the spreading of leishmaniasis agents in the Mediterranean region Euro Surveill 2013 14 30 20540 23929183 10.2807/1560-7917.es2013.18.30.20540

[B2] Berens-Riha N Fleischmann E Pratlong F Bretzel G von Sonnenburg F Löscher T Cutaneous leishmaniasis (Leishmania tropica) in a German tourist after travel to Greece J Travel Med 2009 14 220 222 10.1111/j.1708-8305.2008.00291.x 19538585

[B3] Martín-Sánchez J Pineda JA Morillas-Márquez F García-García JA Acedo C Macías J Detection of Leishmania infantum kinetoplast DNA in peripheral blood from asymptomatic individuals at risk for parenterally transmitted infections: relationship between polymerase chain reaction results and other Leishmania infection markers Am J Trop Med Hyg 2004 14 5 545 548 15155989

[B4] Bailey MS Lockwood DNJ Cutaneous leishmaniasis Clin Dermatol 2007 14 203 211 10.1016/j.clindermatol.2006.05.008 17350500

[B5] Peters N Sacks D Immune privilege in sites of chronic infection: Leishmania and regulatory T cells Immunol Rev 2006 14 159 179 10.1111/j.1600-065X.2006.00432.x 16972903

[B6] Schönian G Nasereddin A Schweynoch C Schallig HD Presber W Jaffe CL PCR diagnosis and characterization of Leishmania in local and imported clinical samples Diagn Microbiol Infect Dis 2003 14 349 358 10.1016/S0732-8893(03)00093-2 12967749

[B7] Jamjoom MB Ashford RW Bates PA Kemp SJ Noyes HA Towards a standard battery of microsatellite markers for the analysis of the Leishmania donovani complex Ann Trop Med Parasitol 2002 14 3 265 270 10.1179/000349802125000790 12061973

[B8] Richter J Hanus I Häussinger D Löscher T Harms G Mucosal Leishmania infantum infection Parasitol Res 2011 14 959 962 10.1007/s00436-011-2356-x 21499751

[B9] del Giudice P Marty P Lacour JP Perrin C Pratlong F Haas H Dellamonica P Le Fichoux Y Cutaneous leishmaniasis due to Leishmania infantum. Case reports and literature review Arch Dermatol 1998 14 2 193 198 10.1001/archderm.134.2.193 9487211

[B10] Svobodová M Alten B Zídková L Dvorák V Hlavacková J Mysková J Seblová V Kasap OE Belen A Votýpka J Volf P Cutaneous leishmaniasis caused by Leishmania infantum transmitted by Phlebotomus tobbi Int J Parasitol 2009 14 2 251 256 10.1016/j.ijpara.2008.06.016 18761342

[B11] Rogers MB Downing T Smith BA Imamura H Sanders M Svobodova M Volf P Berriman M Cotton JA Smith D Genomic confirmation of hybridisation and recent inbreeding in a vector-isolated Leishmania population PLoS Genet 2014 14 1 e1004092 10.1371/journal.pgen.1004092 24453988 PMC3894156

[B12] Morizot G Kendjo E Mouri O Thellier M Pérignon A Foulet F Cordoliani F Bourrat E Laffitte E Alcaraz I Bodak N Ravel C Vray M Grögl M Mazier D Caumes E Lachaud L Buffet PA Travellers with Cutaneous Leishmaniasis Cured Without Systemic Therapy Clin Infect Dis 2013 14 3 370 380 10.1093/cid/cit269 23633111

[B13] Paniz Mondolfi AE Stavropoulos C Gelanew T Loucas E Perez Alvarez AM Benaim G Polsky B Schoenian G Sordillo EM Successful treatment of Old World cutaneous leishmaniasis caused by Leishmania infantum with posaconazole Antimicrob Agents Chemother 2011 14 4 1774 1776 10.1128/AAC.01498-10 21282455 PMC3067187

[B14] Blum J Desjeux P Schwartz E Beck B Hatz C Treatment of cutaneous leishmaniasis among travellers J Antimicrob Chemother 2004 14 2 158 166 10.1093/jac/dkh058 14729756

[B15] Azab AS Kamal MS el-Haggar MS Metawaa BA Hindawy DS Early surgical treatment of cutaneous leishmaniasis J Dermatol Surg Oncol 1983 14 1007 1012 6358304 10.1111/j.1524-4725.1983.tb01055.x

